# Effects of Different Ecological Floating Bed Plant Assemblages on Water Purification and Phytoplankton Community Structure in Shallow Eutrophic Lakes: A Case Study in Lake Taihu

**DOI:** 10.3390/biology14070807

**Published:** 2025-07-03

**Authors:** Yidong Liang, Ting Zhang, Wei Cui, Zhen Kuang, Dongpo Xu

**Affiliations:** 1 National Demonstration Center for Experimental Fisheries Science Education, Shanghai Ocean University, Shanghai 201306, China; liangyidong1228@163.com (Y.L.);; 2 Scientific Observing and Experimental Station of Fishery Resources and Environment in the Lower Reaches of the Changjiang River, Ministry of Agriculture and Rural Affairs, Freshwater Fisheries Research Center, Chinese Academy of Fishery Sciences, Wuxi 214081, China

**Keywords:** ecological restoration, phytoplankton, functional group, eutrophic lakes, ecological floating beds

## Abstract

To explore the effects of different plant combinations in ecological floating beds on water quality purification and phytoplankton community structure in shallow eutrophic lakes, we conducted a survey of phytoplankton communities within ecological floating beds featuring distinct plant combinations in Meiliang Bay, Lake Taihu, during June and August 2021. The study focuses on two combinations: EA (*Canna indica* + *Acorus calamus* + *Phragmites australis*) and ES (*Canna indica* + *Oenanthe javanica* + *Sagittaria sagittifolia*). Results indicated that ecological floating beds significantly improved water quality, with the strongest restoration effects observed in the EA area. Specifically, turbidity was reduced by 47–89%, while chlorophyll a (Chl-a) concentration inhibition rates reached 82% in June and 54% in August. The comprehensive trophic state index (TLI) remained stable at levels indicating slight eutrophication (≤58.6). Phytoplankton community structure shifted from dominance by eutrophic functional groups (primarily FG M) toward greater diversity. In the EA area, the number of dominant functional groups increased from five (control) to six, and the abundance of the key cyanobacteria group (FG M) declined from 18.29% (control) to 7.86%. Redundancy analysis (RDA) revealed temporal changes in driving factors: nutrients were primary in June (explanation rate: 64.7%), while physical factors dominated in August (explanation rate: 51.2%). This study demonstrates that installing ecological floating beds with diverse plant combinations in shallow eutrophic lakes can effectively alter phytoplankton community structure and enhance in situ water restoration. Among the tested combinations, EA (*Canna indica* + *Acorus calamus* + *Phragmites australis*) exhibited the optimal restoration effect. These findings provide a scientific basis for water environment protection and aquatic biological resource restoration in shallow eutrophic lakes.

## Simple Summary

Shallow eutrophic lakes face significant water quality problems due to nutrient pollution. This study aimed to assess how ecological floating beds (EFBs) with different plant combinations improve water quality and alter phytoplankton communities in Lake Taihu’s Meiliang Bay. We specifically tested two plant mixtures, EA (*Canna indica* + *Acorus calamus* + *Phragmites australis*) and ES (*Canna indica* + *Oenanthe javanica* + *Sagittaria sagittifolia*), during summer 2021. Results demonstrated that EFBs significantly enhanced water quality, with the EA combination being most effective. EA reduced turbidity by 47–89% and inhibited chlorophyll a (an algal biomass indicator) by 54–82%, maintaining slightly eutrophic conditions. Phytoplankton communities shifted away from dominance by problematic cyanobacteria (e.g., functional group M abundance decreased from 18.3% to 7.9%) towards greater diversity and more functional groups. Key drivers of the community changed seasonally, shifting from nutrients in June to physical factors in August. This study concludes that installing EFBs, particularly the EA plant combination, is an effective strategy for in situ water restoration in shallow eutrophic lakes, improving water clarity, controlling algal blooms, and fostering healthier phytoplankton communities. These findings provide crucial scientific support for protecting and restoring water environments in such lakes.

## 1. Introduction

Lake eutrophication poses significant global ecological and environmental challenges, leading to detrimental effects on lake ecosystems, such as frequent cyanobacterial blooms, biodiversity loss, community simplification, and declining fishery resources [1]. A defining characteristic of eutrophic freshwater ecosystems is the proliferation of large amounts of phytoplankton, particularly cyanobacteria [2]. As primary producers and the foundation of aquatic food chains, phytoplankton critically influence ecosystem stability through their community structure and dynamics [3,4]. Their rapid responsiveness to environmental changes also positions them as effective water quality indicators. However, abnormal phytoplankton proliferation triggers algal blooms, with harmful algal blooms (HABs) emerging as a significant threat to global inland waters. Since the 2010s, such proliferation events have increased globally, especially in developing Asian and African countries with intensive agricultural fertilizer use. These outbreaks inevitably compromise aquatic ecosystem structure and function, jeopardizing coastal fisheries and tourism [5].

At present, there are four commonly used functional group classification methods: functional group (FG) [6] plant functional type (PFT) [7], morphological functional group (MFG) [8], and morphological-based functional group (MBFG) [9]. Reynolds first proposed the FG classification method, which divided phytoplankton into 31 different FGs [10]. In 2009, Padisák refined and optimized the classification system, identifying 39 FGs that have since been widely employed in lake ecology research [11]. The FG classification method demonstrates higher environmental interpretability and can effectively reveal correlations between phytoplankton communities and aquatic environmental pressures. This method reveals the transition of water bodies from oligotrophic to eutrophic conditions due to human interference, ultimately recovering to oligotrophic states [12]. Phytoplankton FGs have been applied to complex water bodies with intricate basin morphology [13]. A study by Zhu et al. in Hongcheng Lake found that the FG classification method is particularly sensitive to seasonal changes in the water environment [14]. Given the frequent use of the FG classification method in rivers, lakes, and reservoirs [15,16,17,18,19], the present study also employed the method to categorize phytoplankton FGs.

Regarding eutrophication control, traditional physicochemical remediation technologies are difficult to widely implement due to their high costs, short effective duration, and risks of secondary pollution [20]. In contrast, ecological restoration technologies—with ecological floating beds as a representative approach—have demonstrated significant improvements in water quality and biodiversity in cases such as Taihu Lake and Wuli Lake [21,22]. However, the temporal transformation patterns of phytoplankton communities and their driving factors caused by different plant combinations remain unclear. Over the past 20 years, the rapid industrial and agricultural development around Taihu Lake has led to the frequent gathering and outbreak of algae blooms, and the degree of water eutrophication is severe [21]. There is an urgent need for effective governance. Therefore, conducting relevant experiments in Taihu Lake will help to better understand the effect of ecological floating beds on water body restoration and their impact on aquatic organisms in large eutrophic lakes. In this study, restoration experiments were conducted within the fishery habitat restoration and resource conservation demonstration area of Meiliang Bay, Taihu Lake, utilizing floating beds planted with two ecological plant species. By comparing phytoplankton community composition and structural changes between the restored area and an unrestored control area, we explored the response of phytoplankton community structure to these ecological restoration measures. This research aims to compare the effectiveness of two floating bed plant combinations on water quality and phytoplankton structure, providing a scientific and practical basis for managing eutrophic lakes.

## 2. Materials and Methods

### 2.1. Setup of the Study Area and Sample Site

#### 2.1.1. Overview of the Research Topic Experimental Infrastructure

**Figure 1:**
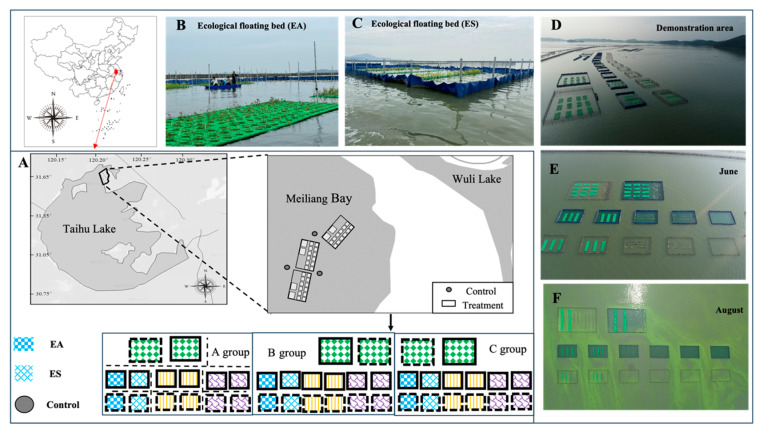
Sampling sites and restoration stages within Taihu Lake, China. Panel (**A**) locations of sampling sites; panels (**B**,**C**) ecological floating beds EA and ES; panels (**D**–**F**) demonstration area during the initial phase (**D**), and during the mid-restoration period in June (**E**) and August (**F**).

Meiliang Bay, located in Wuxi City within the northern section of Taihu Lake (31°28′15″ N, 120°12′40″ E), was chosen as the study location for the experimental system. The bay spans an area of approximately 132 km^2^, with an average water depth of around 2 m. However, it experiences perennial cyanobacterial blooms. Within Meiliang Bay, a designated demonstration area was established for the study, containing three control groups and three parallel treatment experimental groups (Figure 1D). Large and small enclosures were constructed atop the existing lake bed to create controlled environments. These enclosures effectively isolated the internal water bodies from the external ones. The tarpaulin at the bottom was compacted with gabion, reaching the base of the silt layer, thereby ensuring complete separation. The total area of the experimental zone was 6750 m^2^.

#### 2.1.2. Experimental System

The experimental system was established in April 2021. It featured one treatment type with three replicates, and parallel experimental groups were setup. Each experimental group included two ecological floating bed restoration areas, each measuring 15 m by 25 m. These restoration areas were placed within watertight enclosures, maintaining a 1:1 ratio of containment. The biological community within the enclosures was comparable to that in the control area, with no significant differences observed. Additionally, three points were designated as the unrestored control area. The ecological floating bed restoration areas contained the following two combinations: combination 1-EA (*Canna indica* + *Acorus calamus* + *Phragmites australis*) and combination 2-ES (*Canna indica* + *Oenanthe javanica* + *Sagittaria sagittifolia*). Each combination contained 270 pots per plant species (810 pots total per restoration area). In total, there were nine sampling locations, including the EA and ES restoration areas and the control area (as shown in Figure 1). The individual floating beds, after being connected in series by nylon ropes, are all fixed to the stainless-steel piles. Sampling was conducted in June and August 2021 to capture the floating bed plants’ peak growth and active remediation phases post-establishment. These periods align with the optimal seasonal functionality of the selected species, avoiding dormant seasons where treatment effects would be minimal.

### 2.2. Determining Physical and Chemical Parameters of the Water

For each sampling site, water parameters, including temperature (T), dissolved oxygen (DO), and pH, were determined by a HACH multiparameter Water Quality Meter (HACVH, HQ40d, Loveland, CO, USA). The water depth was measured with a hand-held depth sounder (Hondex PS-7, Tokyo, Japan). Simultaneously, the transparency (SD) was measured by a white and black Secchi disk (KCT-8S, Ningbo, Zhejiang, China). Water turbidity (TUR) was measured by a turbidimeter (HANNA, HI98703, Padova, Italy) in situ. Total nitrogen (TN) concentration, total phosphorus (TP) concentration, and chemical oxygen demand (COD_Mn_) were measured using standardized methods and tests [23]. Chlorophyll-a (Chl-a) was extracted using ethanol at 4 °C and expressed as the difference in absorbance between 665 nm and 649 nm using ethanol as the control [24]. To evaluate the trophic state of lakes, the trophic level index (TLI) was calculated based on the concentrations of TP, TN, Chl-a, COD_Mn_, and SD in the water environment [25].

### 2.3. Sample Collection and Identification

Qualitative and quantitative phytoplankton samples were collected at each sampling site to analyze community diversity and composition. For qualitative sampling, phytoplankton were collected by towing a 25# phytoplankton net in an ∞-shaped pattern for 1–3 min at 50 cm below the water surface, with samples preserved in 100 mL bottles using 1–1.5% Lugol’s solution for fixation. For quantitative sampling, 1 L water samples were collected in high-density polyethylene bottles, preserved with 1–1.5% Lugol’s solution, and settled for 24 h; after which the supernatant was siphoned off and the concentrated sediment (20–25 mL) transferred to 50 mL bottles. Microscopic examination involved transferring 0.1 mL of the homogenized quantitative sample to a phytoplankton counting chamber, with species identification and cell counting performed using an Olympus CX31 microscope, where counts were validated if the mean of duplicate measurements per species exhibited ≤15% variation. Species identification referenced the Atlas of Chinese Freshwater Organisms [26] and Atlas of Common Freshwater Phytoplanktonic Algae in China [27], while biomass estimation employed the geometric approximation of the volumetric method by Hillebrand et al. [28].

### 2.4. Data Analysis and Processing

Functional groups (FGs) with a relative biomass greater than 5% were selected as representative FGs. To ensure the data met the assumptions of parametric tests, both phytoplankton FG biomass data and environmental factors were transformed using the logarithmic function lg(x + 1). Pearson correlation analysis was conducted using SPSS 22.0 (IBM Corp., New York, NY, USA) to assess the relationships between variables. Additionally, redundancy analysis (RDA) was performed using R version 4.4.1 to examine the relationships between phytoplankton FGs and environmental factors. All figures were generated using R version 4.3.1 (R Core Team 2023) with the ggplot2 package.

## 3. Results

### 3.1. Environmental Parameters of Water in Various Ecological Restoration Areas

The ecological restoration project has achieved significant improvements in water quality within the Meiliang Bay Demonstration Area. In June and August (Table 1), the water turbidity (TUR) in the restoration areas (EA and ES) decreased by an average of 47% to 89% compared with the control group, with the difference being statistically significant (*p* < 0.05). Notably, in June, the transparency (SD) in the restoration areas increased to four times that of the control group (0.99 m vs. 0.25 m). The key indicator, chlorophyll a (Chl-a), consistently decreased significantly in the restoration areas, with the EA area showing a particularly pronounced inhibitory effect (a decrease of 82% in June and 54% in August). The Comprehensive Nutritional Index (TLI) indicated a significant alleviation of eutrophication in the restoration areas. In the control group, moderate eutrophication was observed in June, and severe eutrophication occurred in August, whereas the EA area remained at a mild level throughout. During the high-temperature period in August, all areas faced deteriorating water quality pressures. In the control group, Chl-a, TUR, and COD_Mn_ increased by 72%, 114%, and 66%, respectively. However, the treated areas maintained the core benefits of lower turbidity, algal inhibition, and improved TLI. Among them, the EA area performed better stably maintaining a low nutritional level (TLI ≤ 58.6) and a low algal biomass (Chl-a ≤ 67.22 μg/L).

### 3.2. Phytoplankton Community Composition and Functional Groups in Different Ecological Restoration Areas

During the survey, a total of 103 species of phytoplankton belonging to seven phyla were identified. The phytoplankton community composition in the demonstration area was categorized as the green algae–diatom–cyanobacteria–cryptophyte type, with green algae being the predominant group. The number of phytoplankton species significantly increased as the restoration time progressed. Following the phytoplankton FG classification method proposed by Reynolds [10,11], a total of 21 FGs were identified in the demonstration area throughout the study period. These FGs were labeled as B, C, D, E, F, G, J, K, Lo, M, MP, N, P, S1, T, W1, W2, X1, X2, Y, and LM. Table 2 provides information on the representative species and habitat characteristics of each FG. The FGs with a relative biomass greater than 5% in at least one sample were considered dominant FGs. The number of dominant phytoplankton FG was determined as 5, 6, and 6 in the Control, EA, and ES areas, respectively. In addition, both the EA and ES areas had more dominant FGs compared with the control area. This suggests that the restoration measures implemented in the EA and ES areas contributed to an increase in the number of dominant phytoplankton FGs, surpassing those in the control area.

**Figure 2:**
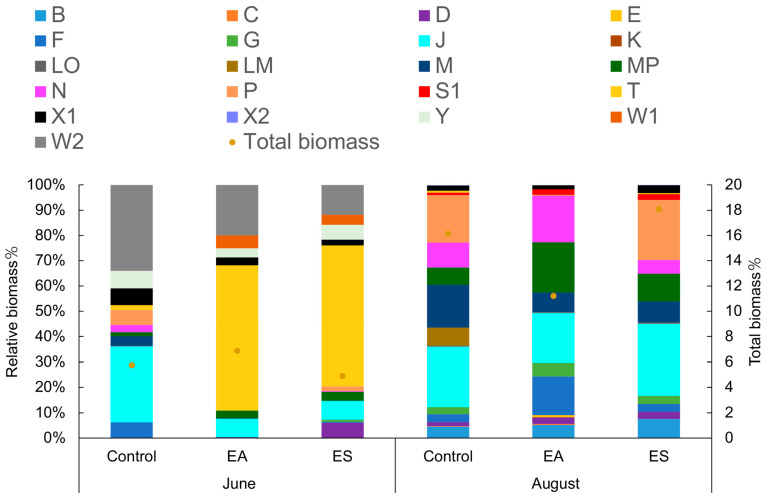
Relative biomass and total biomass of phytoplankton functional groups in different restoration areas in June and August.

The relative biomass and total biomass of the phytoplankton FGs in the demonstration area varied between June and August (Figure 2). In June, the dominant FGs were W2, Y, T, J, and F. However, in August, the dominant FGs shifted to B, J, M, MP, N, and P. Analyzing the relative biomass and total biomass of the phytoplankton FGs in different ecological restoration areas, the dominant FGs in the control area changed from W2, J, LM, Y, X1, P, and F in June to M, MP, N, P, and J in August. The EA area experienced a shift from T, W2, J, and W1 in June to MP, J, N, F, M, and B in August. As for the ES area, the dominant FGs changed from T, W2, and J in June to a different composition in August. Control and M are most dominated by W2. The W2 FG mainly includes *Trachelomonas* sp., *Strombomonas* sp., and *Trachelomonas spinulosa*, and its indicator environment is mesotrophic and shallow water, which is consistent with the real environment. Therefore, the control area FG and W2 have the main advantage. The representative species of the T-functional group are *Planctonema* and *Mougeotia* sp., with a growth strategy of R-type and light as the limiting factor, suitable for growing in continuously mixed water bodies on the surface. The R-type (tolerance) represents the FG that dominates under conditions of abundant material but limited energy. Therefore, due to the influence of the ecological floating bed plants, the proportion of T and FGs is relatively high in the EA and ES regions.

### 3.3. Diversity of Phytoplankton and Ecosystem Stability Across Varied Ecological Restoration Areas

**Figure 3:**
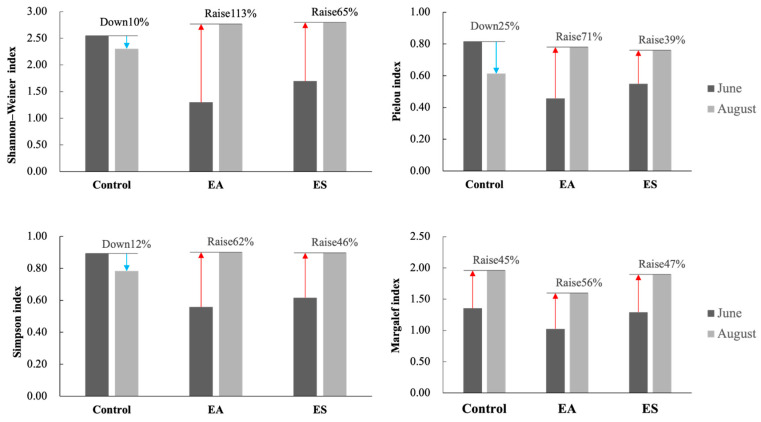
Comparative analysis of changes in phytoplankton diversity between June and August. Data for June are shown in black; August data are shown in gray. Decreases and increases are indicated by blue and red arrows, respectively.

All three biodiversity indices (Shannon–Wiener, Margalef, and Pielou) reflected a polluted state in the demonstration area, aligning with the integrated trophic level index (∑TLI) assessment. Crucially, ecological restoration markedly enhanced diversity metrics. The Shannon–Wiener index in the EA area surged by 113% and by 65% in the ES area from June to August, while the control declined by 10%. The Simpson index rose by 62% in EA and 46% in ES during the same period, contrasting with a 12% drop in the control (Figure 3). Composite analysis confirmed that EA communities achieved superior stability exhibiting the highest gains across all indices and created significantly more favorable conditions for phytoplankton community establishment than ES areas.

### 3.4. Relationship Between Dominant Phytoplankton FGs and Environmental Factors in Different Ecological Restoration Areas

#### 3.4.1. Pearson Correlation Analysis of Dominant Phytoplankton FGs and Environmental Factors

**Figure 4:**
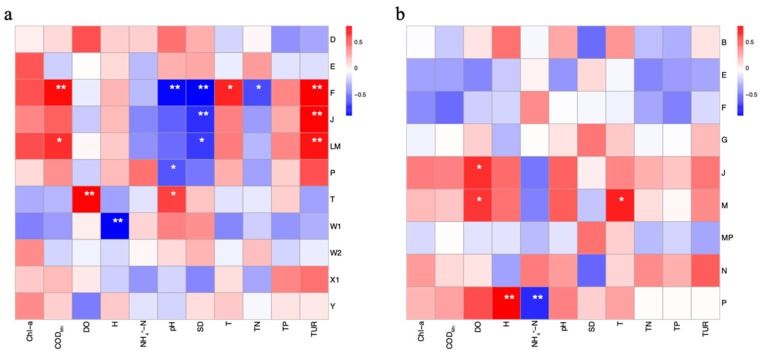
Pearson correlation analysis between the dominant FGs of phytoplankton and water quality evaluation index in Meiliang Bay in June (**a**) and August (**b**), statistical significance is indicated by star marker (* *p* < 0.05, ** *p* < 0.01).

A correlation analysis between the dominant phytoplankton FGs and the environmental factors of water quality in different restoration areas was conducted, and the results are presented in Figure 4. Various FGs exhibited diverse correlations with the environmental factors in the water body. In June, FG and F exhibited significantly positive correlations with TUR (*p* < 0.01), COD_Mn_ (*p* < 0.01), and Temp (*p* < 0.05), while showed negative correlations with TN (*p* < 0.05), SD (*p* < 0.01), and pH (*p* < 0.01). FG and J demonstrated significant correlations with TUR (*p* < 0.01) and SD (*p* < 0.01). FG and LM exhibited significantly positive correlations with TUR (*p* < 0.01) and COD_Mn_ (*p* < 0.05) and a negative correlation with SD (*p* < 0.05). FG and P demonstrated a significantly negative correlation with pH (*p* < 0.05), and FG and T showed significantly negative correlations with pH (*p* < 0.05) and DO (*p* < 0.01). FG and W1 exhibited a significantly negative correlation with H (*p* < 0.01). In August, FG and J showed a significantly positive correlation with DO (*p* < 0.05), FG and M exhibited positive and significant correlations with T and DO (*p* < 0.05), and FG and P demonstrated significantly positive correlations with H (*p* < 0.01) and negative correlations with NH_4_^+^-N (*p* < 0.01).

#### 3.4.2. Redundancy and Canonical Correspondence Analysis of Dominant FGs and Environmental Factors

**Figure 5:**
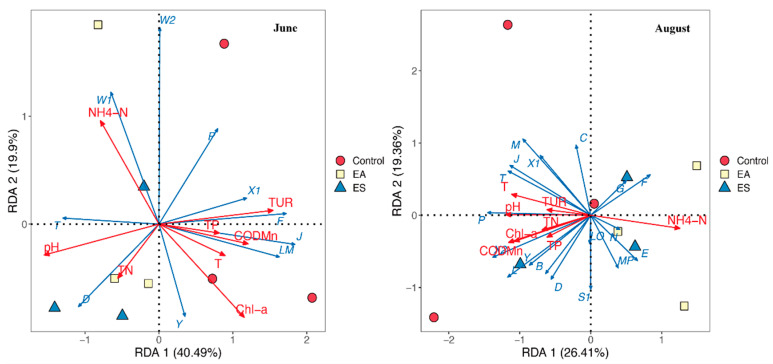
RDA of phytoplankton dominant FGs and environmental factors in different ecological restoration areas in June and August.

As the ecological restoration progressed, the impact of environmental factors on the distribution of phytoplankton communities in different restoration areas was investigated. Initially, the biomass of the dominant FG in June within the experimental area was selected for detrended correspondence analysis (DCA). The resulting length of the ranking axis in June and August were 2.3234 and 1.48622, respectively. Hence, a linear model was employed for RDA, with environmental factors including T, TUR, pH, TN, TP, Chl-a, COD_Mn_, and NH_4_^+^-N. As shown in Figure 5, in June, the environmental factors found to be significant for the phytoplankton FGs in different restoration areas included TUR, Chl-a, NH_4_^+^-N, and pH. In August, the control area primarily clustered in the second three quadrants, with closely related FGs being M, J, and P. The EA and ES areas were more concentrated in the first four quadrants, and their closely related FGs show positive correlations with most of the physical environmental factors. FGs, MP, E, and G exhibited positive correlations with NH_4_^+^-N, while FG F displayed a negative correlation with TUR. The concentration of FGs in EA/ES within quadrants showing positive correlations with multiple environmental factors suggests a shift towards communities adapted to improved water quality conditions, potentially characteristic of a recovering ecosystem. The negative correlation of FG and F (likely sensitive to disturbance) with TUR in these areas further supports this interpretation, indicating lower turbidity stress. The distinct clustering of the control area with FGs, M, J, and P (often associated with different or potentially more disturbed/tolerant conditions) in August reinforces the differences in community structure driven by the restoration measures and the differing environmental pressures.

## 4. Discussions

### 4.1. Composition and Successional Characteristics of Dominant Phytoplankton Functional Groups

In the present study, after implementing in situ remediation measures, the comprehensive nutritional status index evaluation results show that the remediation area significantly decreased compared with the control area; the water quality was also significantly improved, indicating that the introduction of remediation measures has an improvement effect on water quality. The water quality restoration effect was the best in the EA area, with the lowest reduction in TN and TP levels. The distribution patterns of phytoplankton, influenced by lake hydrological conditions and human activities, serve as effective indicators of the ecological integrity and water quality of lakes [29,30]. Throughout the study period, a total of 103 species from seven phytoplankton phyla were identified in the demonstration area. In both June and August, the phytoplankton community comprised 21 FGs, consistent with the findings in Taihu Lake (20 FGs) and Yangcheng West Lake (21 FGs) [31,32]. Among these, 11 dominant FGs were observed, namely, D, E, F, J, LM, P, T, W1, W2, X1, and Y. The number of dominant phytoplankton FGs varied among the different restoration areas. Specifically, the control, EA and ES areas exhibited 5, 6, and 6 dominant FGs, respectively.

Spatiotemporal heterogeneity often leads to different lake water habitats, resulting in the emergence of phytoplankton FGs that match the habitats [33]. Consequently, the placement of restoration measures in different ecological restoration areas results in varying water habitats and corresponding phytoplankton FGs. In terms of temporal variation, the biomass of phytoplankton in June was significantly lower than that in August (*p* < 0.05) as restoration efforts progressed over time. The severity of cyanobacterial blooms was the highest in August, with small cyclophytes (*Cyclotella* sp.) and starry algae (*Asterionella glacialis*) dominating FGs, B, and C, respectively [11,34]. These findings indicate the eutrophication levels in the water bodies of the demonstration area. Specifically, M and FG represented by Microcystis sp., exhibited varying proportions across the different following restoration areas: the control area (18.29%) > EA area (7.86%) > ES area (8.45%). The significantly higher proportion of the M and FG in the control area compared with other restoration areas suggests that different ecological restoration measures have significant effects on managing eutrophic lakes [35]. Overall, the phytoplankton FGs in the demonstration area exhibited a succession from W2 + Y + T + J + F to B + J + M + MP + N + P. These dominant FGs were commonly found in turbid, nutrient-rich waters, indicating the eutrophication of the water in the demonstration area. In the present study, 11 dominant FGs were identified with the FG classification method, of which seven represented eutrophic FGs. This suggests that the habitats within the area were all eutrophic, aligning with the findings of the TLI (∑) water quality assessment. It confirms that the FG classification method is sensitive to environmental changes in water bodies and effectively reflects alterations in the nutrient status of the water. The habitat characteristics indicate by the FG functional groups can accurately reflect the impact of restoration measures [36]. Thus, the FG classification method is suitable for evaluating the ecological control effects of different ecological restoration areas on eutrophic ecosystems.

### 4.2. Key Drivers of Phytoplankton Functional Groups

Phytoplankton are widely distributed and exhibit rapid responses to environmental changes. The specific environmental factors that influence phytoplankton vary across different water bodies [37]. Dissolved oxygen, light, temperature, and nutrient levels are among the key factors directly affecting phytoplankton community composition [38]. In the demonstration area’s various ecological restoration areas, the presence of phytoplankton can alter the water’s environmental conditions, indirectly impacting the phytoplankton community structure. Correlation analysis between the biomass of dominant FGs in different ecological restoration areas and water environmental factors revealed significant effects of TUR, SD, pH, COD_Mn_, and TP on certain FGs in early summer (June). In late summer (August), temperature, NH_4_^+^-N, and DO had a significant impact on some FGs.

Water temperature is crucial for phytoplankton growth and reproduction, as it affects enzymatic reactions in photosynthesis and the intensity of respiration [32]. The higher phytoplankton biomass observed in late summer (August) compared with early summer (June) suggests that increasing water temperature positively influences phytoplankton biomass. Water temperature notably influenced the distribution of FGs M and F, represented by Microcystis sp. FGs J (*Scenedesmus* sp.) and T (*Planctonema*) showed significant positive correlations with water temperature, with the species in these FGs belonging to cyanobacterial and green algal phyla, respectively, which thrive under higher summer temperatures. The pH of the water column is an important factor influencing the growth and metabolism of most phytoplankton, with phytoplankton photosynthesis being inhibited under excessively high or low pH levels [39,40]. The mean pH value in the demonstration area was 8.93, which favors the growth of FG J. Qian et al. also observed significant influences of environmental factors on the distribution characteristics of phytoplankton FGs through long-term studies of phytoplankton FG changes in Poyang Lake [41].

The RDA results clearly demonstrated the dynamic changes in the relationship between phytoplankton community structure and environmental factors in the Taihu demonstration area from June to August. The ecological restoration projects (EA, ES) have significantly improved the local water quality environment (especially reducing the impact of high pollution), resulting in a significant separation of the phytoplankton community structure in the restoration area from that in the unrestored area (control). pH becomes a key driving factor in summer. The EA area demonstrated the ability to maintain a relatively stable community structure or a balanced environment, while the ES area showed a very prominent response to pH changes. The decline in the model’s explanatory power suggests that the role of non-environmental factors may increase in the later stage of seasonal changes. These results, together with the previously mentioned diversity index analysis (such as a 113% increase in the Shannon index in the EA area), collectively demonstrate that the EA restoration project has achieved more substantial ecological restoration effects in the short term (during this study period).

### 4.3. Research Priorities Following Eutrophication Remediation

This study demonstrates significant improvements in water quality and phytoplankton community structure following remediation measures. However, the cascading effects on higher trophic levels, particularly concerning fish biomass and community composition, require further investigation. The observed enhancements—including reduced turbidity, suppression of toxic cyanobacterial blooms, and concurrent shifts in phytoplankton functional groups potentially facilitating zooplankton succession—collectively foster more favorable fish habitat conditions [42,43]. Specifically, diminished organic matter decomposition may alleviate hypoxia risks, thereby expanding suitable habitat for fish. Furthermore, the shift towards phytoplankton functional groups dominated by edible chlorophytes (e.g., the J group) could enhance the quality of the food web base by improving zooplankton resource availability and nutritional value, potentially benefiting planktivorous fish through trophic bottom-up effects [44,45,46].

While this study provides robust evidence for short-term improvements in eutrophic water bodies, long-term monitoring is essential to evaluate the sustainability of restoration, potential ecosystem feedback mechanisms, and ongoing management needs. Future research should prioritize the following: (1) multi-seasonal and interannual monitoring programs; (2) quantitative assessment of ecological benefits relative to maintenance costs; and (3) systematic tracking of fish population dynamics (biomass, abundance, community structure) and concomitant changes in food web architecture. Such comprehensive approaches will enable quantitative evaluation of restoration benefits for higher trophic levels, ensuring that improvements achieved at the water quality and primary producer levels effectively translate to enhanced fish community health and overall ecosystem function.

## 5. Conclusions

A total of 103 species of phytoplankton from 7 phyla were identified in the demonstration area and classified into 21 functional groups (FGs), among which 11 were dominant groups. Different ecological restoration measures can significantly enhance the diversity of phytoplankton species and dominant functional groups in eutrophic lakes, reduce the degree of eutrophication, and stabilize water quality. RDA indicated that turbidity (TUR), transparency (SD), dissolved oxygen (DO), pH, and permanganate index (COD_Mn_) were significantly correlated with the biomass of dominant functional groups. All biological algae control methods have significant improvement effects on water quality and the structure of plankton communities. Among them, the ecological floating bed combination EA (*Canna indica* + *Acorus calamus* + *Phragmites australis*) has a better effect than combination ES (*Canna indica* + *Oenanthe javanica* + *Sagittaria sagittifolia*). The FG functional group classification method is applicable to evaluate the ecological regulation efficacy of ecological restoration measures on eutrophic lakes, providing a scientific basis for governance practices.

## Figures and Tables

**Table 1:** Water environmental factors of different ecological restoration areas in June and August.

Time	Group	Control	EA	ES
June	T (°C)	28.23 ± 0.40 ^a^	27.43 ± 0.12 ^b^	27.30 ± 0.36 ^b^
	pH	7.99 ± 0.15 ^c^	9.64 ± 0.08 ^a^	9.69 ± 0.16 ^a^
	DO (mg/L)	9.65 ± 0.08 ^a^	9.60 ± 0.55 ^a^	10.04 ± 1.10 ^a^
	H (m)	2.20 ± 0.00 ^a^	2.17 ± 0.05 ^a^	2.20 ± 0.00 ^a^
	TUR (NTU)	41.33 ± 1.67 ^a^	5.45 ± 0.94 ^b^	4.73 ± 0.82 ^b^
	SD (m)	0.25 ± 0.02 ^a^	0.99 ± 0.11 ^b^	0.99 ± 0.14 ^b^
	TN (mg/L)	7.61 ± 0.86 ^a^	6.34 ± 1.25 ^b^	7.02 ± 0.00 ^a^
	TP (mg/L)	0.15 ± 0.03 ^a^	0.06 ± 0.09 ^a^	0.08 ± 0.01 ^b^
	NH_4_^+^-N (mg/L)	0.22 ± 0.18 ^a^	0.23 ± 0.10 ^a^	0.39 ± 0.05 ^a^
	Chl-a (ug/L)	85.60 ± 4.05 ^a^	15.57 ± 11.45 ^c^	48.77 ± 14.32 ^b^
	COD_Mn_ (mg/L)	5.01 ± 0.23 ^a^	3.54 ± 0.08 ^a^	4.09 ± 0.41 ^a^
	TLI	69.8	57.3	59.4
	Trophic state	MOD	LIG	LIG
August	T (°C)	30.87 ± 0.12 ^a^	30.37 ± 0.09 ^b^	30.37 ± 0.12 ^b^
	pH	8.30 ± 0.05 ^a^	8.14 ± 0.05 ^b^	8.12 ± 0.06 ^b^
	DO (mg/L)	13.98 ± 0.75 ^a^	12.10 ± 0.56 ^b^	12.22 ± 0.33 ^b^
	H (m)	2.67 ± 0.05 ^a^	2.60 ± 0.00 ^a^	2.63 ± 0.05 ^a^
	TUR (NTU)	88.50 ± 21.84 ^a^	49.07 ± 15.92 ^bc^	54.10 ± 21.89 ^b^
	SD (m)	0.27 ± 0.02 ^a^	0.30 ± 0.03 ^a^	0.32 ± 0.01 ^a^
	TN (mg/L)	1.99 ± 0.40 ^a^	1.44 ± 0.44 ^a^	1.54 ± 0.15 ^a^
	TP (mg/L)	0.19 ± 0.04 ^a^	0.14 ± 0.07 ^a^	0.16 ± 0.06 ^a^
	NH_4_^+^-N (mg/L)	0.29 ± 0.09 ^a^	0.42 ± 0.07 ^a^	0.35 ± 0.06 ^a^
	Chl-a (ug/L)	147.35 ± 33.86 ^a^	67.22 ± 14.46 ^b^	97.26 ± 20.82 ^ab^
	COD_Mn_ (mg/L)	8.31 ± 1.60 ^a^	5.74 ± 0.84 ^a^	6.65 ± 0.86 ^a^
	TLI	70.2	58.6	60.4
	Trophic state	SEV	LIG	MOD

Note: After the restore treatment, differences among different groups were indicated by letter notation (a, b, c; groups with the same letter indicates no significant difference, *p* > 0.05). SEV: severe; LIG: light; MOD: moderate.

**Table 2:** Functional classification of phytoplankton in Meiliang bay and their suitable habitats.

Functional Group	Representative Species (Genus)	Habitat Characteristics
B	*Cyclotella* sp., *Stephanodiscus* sp., *Cyclotella ocellata*, *Cyclotella bodonica*, *Cryptomonas marssonii*, *Cryptomonas ovata*, *Cryptomonas erosa*	Eutrophic, large, deep, or shallow water bodies
C	*Asterionella glacialis*	Mesotrophic, medium to small-sized water bodies
D	*Synedra* sp., *Synedra acus*, *Synedra tabulata*, *Nitzschia* sp., *Nitzschia microcephala*	High-nutrient content, low transparency
E	*Dinobryon* sp.	Oligotrophic, mixed, shallow water
F	*Dictyosphaerium* sp., *Sphaerocystis* sp., *Oocystis* sp., *Oocystis lacustris*, *Kirchneriella* sp., *Nephrocytium agardhianum*, *Micractinium pusillum*, *Treubaria* sp.	Eutrophic, clean, strong water mixing
G	*Pandorina* sp., *Eudorina* sp.	Eutrophic, stagnant water bodies
J	*Scenedesmus* sp., *Scenedesmus acuminatus*, *Scenedesmus quadricanda*, *Antinomy quadricanda*, *Scenedesmus dimorphus*, *Tetrastrum* sp., *Coelastrum* sp., *Pediastrum* sp., *Actinastrum* sp., *Tetraedronoidrae*, *Pediastrum simplex*, *Pediastrum duple*, *Crucigenia tetrapedia*, *Crucigenia apiculata*, *Crucigenia. Quadrata*, *Tetrastrum* sp., *Tetraedrum heterocanthum*, *Scenedesmus platydiscus*, *Crucigenia fenestrata*, *Pediastrum tetras*, *Selenastrum bibraianum*	Mesotrophic to eutrophic shallow water bodies
K	*Aphanocapsa* sp.	Eutrophic shallow water bodies
Lo	*Merismopedia* sp., *Chroococcus* sp., *Ceratium* sp., *Peridinium* sp.	Mesotrophic to eutrophic, medium to large-sized water bodies, variable depth
M	*Microcystis* sp.	Relatively stable mesotrophic to eutrophic water bodies, moderate transparency
MP	*Pseudanabaena catenata*, *Oscillatoria* sp., *Navicula* sp., *Cocconeis* sp., *Gomphonema* sp., *Achnathes* sp., *Navicula capitata*, *Navicula radiosa*	Frequently disturbed, turbid shallow lakes
N	*Cosmarium* sp., *Staurastrum* sp.	Continuously or semi-continuously mixed water bodies
P	*Closterium* sp., *Melosira* sp., *Fragilaria* sp., *Melosira granulate*	Mesotrophic to eutrophic, continuously or semi-continuously mixed water bodies
S1	*Phormidium* sp., *Planktothrix* sp., *Rhabdogloea*	Mesotrophic to eutrophic, mixed water bodies, low transparency
T	*Planctonema*, *Mougeotia* sp.	Uniformly mixed water bodies with thermocline
W1	*Euglena* sp., *Phacus* sp., *Phacus hamatus*, *Phacus longicauda*, *Euglena acus*	Organically polluted, shallow water
W2	*Trachelomonas* sp., *Strombomonas* sp., *Trachelomonas spinulosa*	Mesotrophic, shallow water
X1	*Schroederia* sp., *Ankistrodesmus* sp., *Chlorella* sp., *Ankistrodesmus spiralis*, *Ankistrodesmus falcatus*, *Monoraphidium*	Highly mixed eutrophic shallow water bodies
X2	*Chroomonas* sp., *Chroomonas acuta uterm*, *Chroomonas acuta*	Highly mixed meso-eutrophic shallow water bodies
Y	*Gymnodinium* sp., *Cryptomonas* sp., *Komma* sp.	Stagnant water environments, mesotrophic to eutrophic
L_M_	*Ceratium hirundinella*	Eutrophic to hypereutrophic, medium-sized water bodies

## Data Availability

The data referenced in this article is included within the text.
